# Insights Into Leukocyte Trafficking in Inflammatory Arthritis – Imaging the Joint

**DOI:** 10.3389/fcell.2021.635102

**Published:** 2021-03-09

**Authors:** Julia E. Manning, Jonathan W. Lewis, Lucy-Jayne Marsh, Helen M. McGettrick

**Affiliations:** Rheumatology Research Group, Institute of Inflammation and Ageing, University of Birmingham, Birmingham, United Kingdom

**Keywords:** imaging, leukocyte, adhesion, migration, arthritis

## Abstract

The inappropriate accumulation and activation of leukocytes is a shared pathological feature of immune-mediated inflammatory diseases (IMIDs), such as rheumatoid arthritis (RA) and psoriatic arthritis (PsA). Cellular accumulation is therefore an attractive target for therapeutic intervention. However, attempts to modulate leukocyte entry and exit from the joint have proven unsuccessful to date, indicating that gaps in our knowledge remain. Technological advancements are now allowing real-time tracking of leukocyte movement through arthritic joints or *in vitro* joint constructs. Coupling this technology with improvements in analyzing the cellular composition, location and interactions of leukocytes with neighboring cells has increased our understanding of the temporal dynamics and molecular mechanisms underpinning pathological accumulation of leukocytes in arthritic joints. In this review, we explore our current understanding of the mechanisms leading to inappropriate leukocyte trafficking in inflammatory arthritis, and how these evolve with disease progression. Moreover, we highlight the advances in imaging of human and murine joints, along with multi-cellular *ex vivo* joint constructs that have led to our current knowledge base.

## Overview of the Leukocyte Recruitment Cascade

**FIGURE 1:**
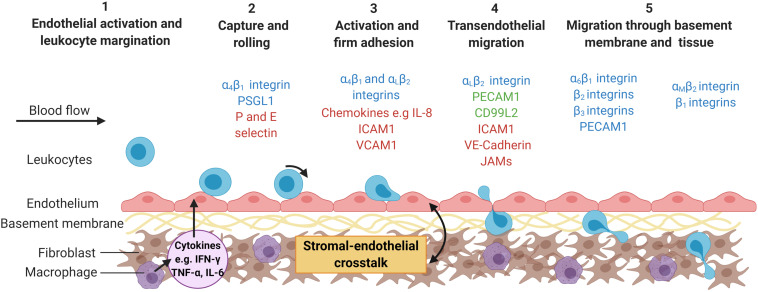
The multi-step leukocyte adhesion cascade. Infection or tissue damage causes the localized release of danger signals, including cytokines, which (1) activate the endothelium causing the expression of capture receptors (P- or E-selectins), adhesion molecules (IgSF members – ICAM-1; VCAM-1) and presentation of chemokines or lipids on their surface (2) (Zhang et al., 2011). Marginated leukocytes are captured, roll along the endothelial surface until (3) they receive an activation signal (typically chemokine mediated) resulting in inside-out signaling and integrin activation (α_L_β_2_-integrin; α_4_β_1_-integrin) (Ley et al., 2007). This allows for stabilization of the adhesive interactions (α_L_β_2_-ICAM-1 and α_4_β_1_-VCAM-1 interactions) and cytoskeletal rearrangement in the leukocyte allowing (4) them to crawl over the endothelium, migrate across the endothelium (ICAM-1; JAM-1; CD31; VE-cadherin) and into the subendothelial space (Ley et al., 2007), (5) where they encounter the basement membrane and stromal compartment. This process is tightly regulated by the haemodynamic forces of the flowing blood and the stromal derived signals experienced by the endothelium (McGettrick et al., 2012). Some of the major molecules involved in this process are represented in the schematic, with molecules colored according to cell type expressing them; leukocytes = blue; endothelial cells (ECs) = red and expressed by both leukocytes and ECs = green. ICAM-1, Intracellular adhesion protein 1; IL-8, interleukin-8; JAM, junctional adhesion molecule; PECAM-1, platelet endothelial cell adhesion molecule 1; PSGL1, P-selectin glycoprotein ligand-1; VE-cadherin, vascular endothelial cadherin; VCAM-1, vascular adhesion protein 1. Created with BioRender.com.

Over the last 30 years, the general understanding of how leukocytes migrate out of the blood, across the endothelium and through inflamed tissues has been extensively researched providing us with a step by step cascade of events (Figure 1) (Ley et al., 2007; Vestweber, 2015). In line with this, imaging techniques for analyzing the individual steps of the cascade, from leukocyte capture through to migration into the tissue, have improved exponentially giving us much more granularity on the temporal and dynamic kinetics of these events and the key molecules involved. Chronic inflammatory arthritis is characterized by the aberrant accumulation and activation of such leukocytes within the synovial tissue, along with tissue-resident stromal cells (fibroblasts) becoming epigenetically reprogrammed, both of which drive tissue and bone damage leading to pain and immobility in patients with, for example, rheumatoid arthritis (RA) or psoriatic arthritis (PsA). The clinical urgency to identify new drug targets that limit the trafficking of pathogenic leukocytes and promote the entry of regulatory cells into chronically inflamed tissues, such as the joint, are partly responsible for driving forward the imaging techniques necessary to visualize these processes in real-time. Here, we explore our current understanding of the mechanisms leading to inappropriate leukocyte trafficking in inflammatory arthritis, and how these evolve with disease progression. We provide a historic overview of the imaging techniques that have led to our current knowledge base – considering our progression from 2-D imaging of tissue sections; through to *in vitro* adhesion and migration assays on sections, purified proteins or single-cell layers; into the realm of real-time imaging of 3-D multicellular *ex vivo* joint constructs or whole joints, and beyond. For each technique we will highlight the key kinetics, leukocyte subpopulations and molecules discovered and how these advanced our understanding of process-driven pathology of inflammatory arthritis.

## Imaging Synovial Tissue – Early Insights Into Pathology

**FIGURE 2:**
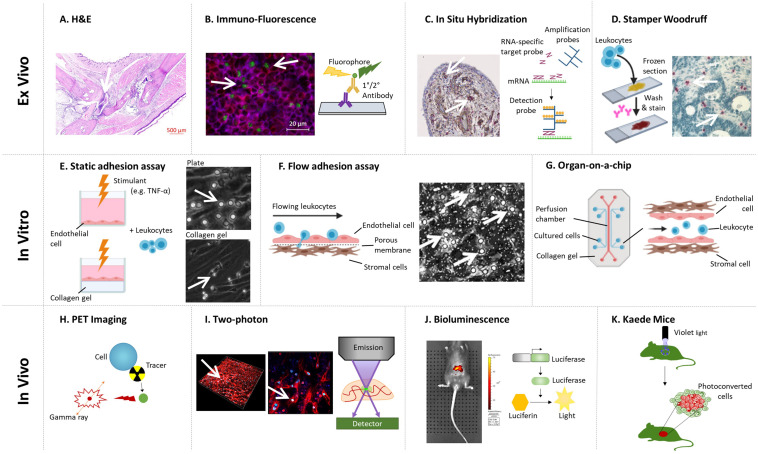
Methodologies for imaging leukocyte infiltration. **(A)** H&E staining (Tucker et al., 2016): decalcified joints are paraffin embedded, sectioned, and stained with H&E to identify leukocyte infiltration. **(B)** Immuno-fluorescence (Honvo-Houéto and Truchet, 2015; JoVE, 2021): tissue sections are stained with fluorescently tagged antibodies against specific proteins to visualize cellular localization using a fluorescent microscope. Representative image shows a lymph node stained for CD3^+^ (Red), CD4^+^ (Blue), and FoxP3^+^ (Green) T-cells. In **(A,B)**, images courtesy of Miss Jenefa Begum (University of Birmingham). **(C)** *In Situ* Hybridization (Menon et al., 2017): an RNA-specific target probe is added to tissue sections, where it hybridizes to its corresponding gene. Amplification probes are added to enhance the detection, which are bound by detection probes. Detection probes can either be fluorescent-, antibody- or radio- labeled. Image of arthritic joint replacement tissue section stained for PDGFRβ (Red), nuclei (Pale Blue), and CD90 (Brown), courtesy of Dr. Triin Major (University of Birmingham). **(D)** Stamper–Woodruff assay (Hirata et al., 2004): leukocytes are added to frozen tissue sections on slides and rotated for 30 min. Non-adherent cells are washed off and the section stained with nuclear dye and/or antibodies of choice. Image courtesy of Dr. Patricia Lalor and Dr. Sarah Edwards (University of Birmingham, Edwards et al., 2006). **(E,F)** Static and flow based adhesion assays (Butler et al., 2009): Endothelial cells or the ligand of interest are seeded onto the well, a filter or onto a collagen gel and treated with stimulant(s) of choice (e.g., TNF-α) for the desired amount of time. Stromal cells can be cultured beneath the endothelium to assess how they regulate endothelial recruitment of leukocytes (Munir et al., 2015; Mcgettrick et al., 2017). **(E)** Leukocytes are added to the well and left to adhere under static conditions, before non-adherent cells washed off and remaining adherent cells imaged via phase contrast microscopy. Image courtesy of Miss Sophie Hopkin (University of Birmingham). **(F)** Leukocytes are perfused across the surface (e.g., endothelial cells or purified protein/carbohydrate) at a wall shear rate determined by the flow rate of a syringe pump. In **(E,F)**, adherent cells appear circular and phase bright, as they become activated and migrate they change morphology and appear phase dark once underneath the endothelial monolayer. **(G)** Organ-on-a-chip (Rothbauer et al., 2020): a micro-scale system to mimic the environment within the human body, combining cultured cells and microfluidic systems – where leukocytes are perfused through a channel coated with endothelial cells, with pericytes and stromal cells cultured on the opposite (basal) side to the endothelial cell. **(H)** PET imaging (White et al., 2017; Momcilovic et al., 2018): cells are tagged using a radioactive tracer that emits a small quantity of Gamma rays. Tagged cells are injected into participants and the location of cells can be tracked and imaged. **(I)** Two photon microscopy (Chen et al., 2017; Cai et al., 2019): fluorescently labeled cells are visualized and their movement tracked in real-time with two-photon microscope either *in vivo* and *ex vivo*. Image of human liver tissue perfused with wheat germ agglutinin and Hoechst and imaged *ex vivo*, courtesy of Dr. Scott P. Davies (University of Birmingham). **(J)** Bioluminescence (Lim et al., 2009): cell specific luciferase knock-in lines are visualized using the whole animal *in vivo* imaging system (IVIS), allowing the tracking of endogenous or injected cells. Image shows 1,1′-dioctadecyltetramethyl indotricarbocyanine Iodide tagged bone marrow mesenchymal stem cells accumulation following injection into wildtype mice; courtesy of Dr. Lozan Sheriff (University of Birmingham). **(K)** Kaede mice (Porter et al., 2018; Steele et al., 2019): UV light illumination of tissues (e.g., draining lymph nodes) *in situ* photoconverts cells from green to red fluorescence. The movement of red fluorescent cells to, and their interaction with cells at, distant tissues can be tracked. In **(A–F,I)**, arrows denote leukocytes on the representative images. Created with BioRender.com.

As early as the 1960’s researchers used haematoxylin and eosin (H&E) to stain tissue sections to visualize tissue architecture (Campbell-Thompson et al., 2012) (Figure 2A) and cellular accumulation in the joints from patients with RA or PsA (Muirden and Senator, 1968; Muirden and Mills, 1971). Time-course experiments in murine models of inflammatory arthritis revealed the accumulation of leukocytes peaked 1–2 days after onset of adjuvant-induced arthritis (AIA) model (Szántó et al., 2004; Gonçalves et al., 2020), day 10 in the KRN serum transfer induced arthritis (STIA) model (LaBranche et al., 2010); and 21 days after immunization in the collagen-induced arthritis (CIA) model (Knoerzer et al., 1997). Similarly, H&E revealed a substantial number of leukocytes accumulating in the synovium of patients with early (disease duration less than 1 year) and established RA (1.5–20 years), with the degree of infiltration correlating with disease activity rather than symptom duration (Baeten et al., 2000). For both human and murine studies, H&E analysis of synovium has highlighted the significant role of tumor necrosis factor-α (TNFα) in driving leukocyte infiltration into the inflamed joint: with TNFα inhibitors (TNFi) reducing leukocyte numbers in the joint leading to reduced clinical scores, which were not seen in non-responders (Williams et al., 1992; Baeten et al., 2000; Barrera et al., 2001), as well as reducing the expression of chemokines within the synovium (Taylor et al., 2000). Similarly, H&E revealed a potential therapeutic benefit of inhibiting chemokine interactions in AIA, where short-chain peptides modeled on the chemokine glycosaminoglycan binding domain of CXCL8 (interleukin-8) decreased leukocyte accumulation and the overall inflammatory score in treated joints (McNaughton et al., 2018). Whilst H&E analysis of tissue sections is an integral outcome measure in almost all studies, it is unable to provide any insight into the specific subsets of leukocytes infiltrating the joint, how these evolve with disease or the mechanisms underpinning their accumulation.

Advances in immunohistochemistry (IHC) and subsequently fluorescence microscopy and *in situ* hybridization (ISH) have expanded our ability to identify individual leukocyte subsets in synovial tissue sections (Menon et al., 2017; Traylor-Knowles, 2018) (Figures 2B,C), furthering our understanding of the composition of leukocytes within the inflamed joint in the various murine models of inflammatory arthritis and human arthritides. For example, monocytes and CD4 T-cell infiltration peaked 2–3 days following onset of AIA, followed by an influx of CD8 T-cells and B-cells which peaked at day 7 (Verschure et al., 1989). Moreover, monocytes and macrophages were observed in the sublining layer of the AIA joint, whilst T and B-cells were located in perivascular clusters and some in small isolated groups (Dijkstra et al., 1987). Indeed, elevated numbers of CD3^+^ T-cells, CD20^+^ B-cells, and CD68^+^ macrophages, along with a concomitant decrease in the numbers of regulatory T-cells (Tregs) were also observed using IHC in synovial biopsies from RA patients with high disease activity scores compared to patients with lower disease activity or in remission (Zhang et al., 2005; Behrens et al., 2007; Alivernini et al., 2017). Moreover, anti-TNFα therapy reduced the numbers of CD3^+^ T-cells, CD22^+^ B-cells, and CD68^+^ macrophages in the joints of patients with RA over a 2 weeks period (Taylor et al., 2000). Indeed, the combination of CD20, CD138 (marking plasma cells), CD3, and CD68 revealed 3 distinct leukocyte profiles (pathotypes) in the synovium of patients with early and established RA based on the absence of leukocytes (pauci-immune), diffuse myeloid infiltrate or the formation of lympho-myeloid aggregates (Humby et al., 2019; Nerviani et al., 2020). Agreeing with earlier studies that described pauci-immune; diffuse and follicular infiltrates (Wagner et al., 1998; Pitzalis et al., 2013). Crucially these different leukocyte synovial pathotypes impact on a patient’s response to therapy – where over 80% of patients with a lympho-myeloid or diffuse myeloid pathotype showed a decrease in their disease activity (DAS28 score) following treatment with the anti-TNFα drug, certolizumab-pegol, whilst less than 30% of patients with the pauci-immune pathotype responded (Nerviani et al., 2020). Such studies reveal two important insights – firstly leukocyte pathotypes highlight the existence of different leukocyte recruitment and retention signatures in subgroups of RA patients. Secondly, although the exact molecular mechanisms for these signatures remain undetermined, TNFα clearly plays a major role in the two pathotypes where elevated levels of leukocytes are observed.

Early IHC and ISH studies revealed for the first time the expression pattern of adhesion molecules and chemokines within arthritic joints (Johnson et al., 1993; Salmi et al., 1997; Matsui et al., 2001; Wang et al., 2004). Macroscopically, expression of certain adhesion molecules (selectins and VCAM-1) have been reported to be restricted to a subpopulation of synovial vessels in patients with chronic arthritis; whilst others [intracellular adhesion molecules 1 and 2 (ICAM-1 and ICAM-2)] were ubiquitously expressed (Salmi et al., 1997). In contrast, little E-selectin was detected on vessels in PsA (Veale et al., 1993). Synovial blood endothelial cells located near or within tertiary lymphoid structures appear to acquire a high endothelial vessel (HEV)-like phenotype with the expression of peripheral node addressin (PNAd), an adhesion molecule normally restricted to HEV’s, observed on synovial endothelial cells in some, but not all, RA and PsA sections analyzed (Salmi et al., 1997; Cañete et al., 2007) – and which may account for the lympho-myeloid/follicular pathotype described (Wagner et al., 1998; Pitzalis et al., 2013). Similarly, rheumatoid synovial endothelium abnormally expresses high levels of the mucosal specific vascular adhesion protein-1 (VAP-1) (Salmi et al., 1997) – indicating that the synovial endothelium acquires the capacity to support the trafficking of gut-homing T-cells during RA pathology. Crucially, the activation status of synovial endothelial cells is sensitive to anti-TNFα therapy, with reduced expression of E-selectin (Paleolog et al., 1996; Tak et al., 1996) and vascular cell adhesion protein 1 (VCAM-1) (Tak et al., 1996) reported in the rheumatoid synovium following treatment – almost certainly accounting for the improvement in disease activity seen in patients with leukocyte rich pathotypes (Nerviani et al., 2020).

In 1976 Stamper and Woodruff transformed tissue analysis from spatial 2-D phenotypic studies to 3-D functional assays (Hirata et al., 2004) (Figure 2D), by allowing lymphocytes to adhere to fixed murine lymph node tissue sections before visualizing their interactions using methyl-green-thionin and light microscopy (Stamper and Woodruff, 1976). Subsequently, groups adapted the Stamper–Woodruff (S–W) assay and began assessing the molecular mechanisms supporting leukocyte adhesion to arthritic synovial tissue. For example, significantly more monocytes adhered to RA synovium compared to other tissues analyzed (foreskin, placenta, and inflamed tonsils), and blocking the capture receptors P-selectin and E-selectin reduced this by >90% and 20–50%, respectively (Grober et al., 1993). Similarly, HL-60 cell (a neutrophil-like leukaemic cell line) adhesion to frozen sections of RA synovium was significantly reduced following treatment with an anti-E-selectin function-blocking antibody or the TNFα inhibitor certolizumab pegol (Shu et al., 2012). Thus demonstrating a pivotal role TNFα, P- and E-selectin in supporting neutrophil and monocyte recruitment to the RA synovium. Furthermore, Salmi et al. (1997) revealed for the first time mucosal lymphocytes bind to VAP-1 expressed by RA synovial vessels, further supporting the concept that gut-specific trafficking address-codes for lymphocytes are hijacked by the rheumatoid joint. Indeed, the mucosal microbiome is now believed to play a crucial role in lymphocyte trafficking outside the inflamed joint, and is highly likely to influence the phenotype of the cells recruited to the joint (reviewed by Buckley and McGettrick, 2018; Manasson et al., 2020; Xu et al., 2020). This functional analysis of tissue sections allowed for the first time mechanistic studies on the proportion of leukocytes binding with or without interventions to be analyzed. Further studies using tissues from other inflammatory arthritides and at different phases of disease are now required to distinct shared and unique features across arthritides, and how these evolve with disease progression.

## Dissecting the Molecular Mechanisms Supporting Aberrant Leukocyte Trafficking – the Creation of *In Vitro* Joint Constructs

The vast majority of our understanding on the dynamics of leukocyte recruitment has been discovered by visualizing cell–cell interactions *in vitro* using assays that incorporate purified proteins or different tissue-resident cells (e.g., blood vascular endothelial cells or fibroblasts) in either 2-D or 3-D formats, and in some cases that also mimic blood flow (Butler et al., 2009; Mcgettrick et al., 2017). These *in vitro* static or flow-based adhesion/migration assays enable the real-time imaging of leukocyte behavior as they interact with the substrate, including their motility (Figures 2E,F). As such they have been a vital tool in aiding our understanding of the molecular processes governing disease onset and perpetuation in many arthritides, especially when patient cells or pharmacological agents have been assessed.

### 2-Dimensional Models

Surprisingly few studies have visualized each step of the adhesion cascade using leukocytes isolated from patients with inflammatory arthritis and models mimicking the joint that incorporate endothelial cells alone or with relevant stromal components (Figures 2E,F). For example, more T-cells adhered and migrated through inflamed endothelium *in vitro* when they were isolated from RA (Taylor et al., 2000) or PsA (Dunky et al., 1997) patients compared cells from the control group – this was thought to be mediated by α_L_β_2_-integrin. Interestingly, the adhesive capability of T-cells appeared to be unaffected by whether a patient exhibits active or inactive RA, with similar numbers of fluorescently labeled T-cells observed binding to either resting or IL-1β stimulated endothelial cells over a 6-h timeframe (Mertens et al., 1994). Similarly, greater numbers of effector memory CD4^+^ T-cells from RA patients migrated further into collagen gels over 48 h following *in vitro* anti-CD3^+^ CD28-induced activation compared to cells isolated from healthy controls (Shen et al., 2017). Combining these data with IHC and metabolite quantification revealed that T-cells from these clinically active RA patients had reduced glycolytic flux that resulted in the overexpression of TSK5, a podosome scaffolding protein found at the leading edge of the cells causing enhanced migratory capacity *in vitro* (Shen et al., 2017). This was one of the first studies to highlight the importance of disease-induced metabolic changes in leukocytes on their adhesive and migratory potential. Similar observations have been linked to genes associated with increased risk of developing RA – for example, super-resolution studies revealed that the expression of the PTPN22 variant (PTPN22W) increased α_L_β_2_-integrin clustering in T-cells resulting in them being more sticky (Burn et al., 2016). Genetic risk factors and abnormal cellular metabolism promote the adhesive and migratory properties of T-cells in patients with RA (Weyand et al., 2020), and potentially other inflammatory arthritides by altering the expression levels or cellular localization of α_L_β_2_-integrin. Whilst directly targeting α_L_β_2_-integrin is not a viable treatment option [see review on leukocyte adhesion deficiency (Harlan, 1993; Kuijpers et al., 1997)], targeting the processes regulating its expression and distribution may provide alternative treatment options for arthritides.

TNFα signaling, itself, can induce leukocytes to shed β_2_-integrins (Gjelstrup et al., 2010), resulting in increased plasma soluble β_2_-integrin (sCD18) concentrations that bind to ICAM-1 on the endothelium to reduce the availability of this molecule to leukocytes, thus reducing adhesive interactions (Kragstrup et al., 2014). However, this homeostatic regulatory pathway is lost in patients with RA and spondyloarthritis (SpA) (Kragstrup et al., 2014), where patients with high disease activity scores have lower plasma sCD18 compared to OA, and thus the potential for elevated levels of leukocyte trafficking (Gjelstrup et al., 2010). Furthermore, B-cells from patients with RA have lost their ability to respond to the adipokine, adiponectin, and therefore their ability to release the peptide hormone, PEPITEM, which acts to limit T-cell migration into inflamed tissues (Chimen et al., 2015). So in addition to lymphocytes acquiring pro-migratory traits, there is also a loss of tonic regulators that normally act to limit tissue infiltration in patients with RA and SpA.

Considering other leukocyte subtypes, patients with RA have higher levels of “reverse migrated” neutrophils in their circulation, suggesting that these cells have sampled the tissue microenvironment and re-entered the circulation across the blood vascular endothelial cells rather than through the lymphatics (Buckley et al., 2006). Moreover, expression of RA susceptibility gene, PTPN22W, enhanced neutrophil migration across TNFα-activated endothelium *in vitro* (Bayley et al., 2015) and may account for the rapid transit of neutrophils through the rheumatoid joint into the synovial fluid. RA neutrophil chemotaxis toward the synovial fluid rich CXCL8 (IL-8) was blocked with the Janus kinase inhibitors (JAKi) (Mitchell et al., 2017), along with reduced neutrophil and T-cell numbers observed in the joints of RA patients treated with barictinib (Tanaka et al., 2018) – suggesting JAKi have potential therapeutic benefit by limiting leukocyte migration in arthritic patients. The higher expression the α_M_ subunit of the α_M_β_2_-integrin on monocytes from RA patients contributed to their enhanced adhesion to resting (uninflamed) and IL-1β stimulated endothelium *in vitro* when compared to the levels observed for monocytes from normal controls (Lioté et al., 1996). By contrast, fewer fluorescently labeled monocytes migrated through TNFα activated human dermal microvascular endothelial cells when they were isolated from PsA patients, compared to monocytes from patients with osteoarthritis, fibromyalgia or type-2-diabetes (as controls) – most likely due to a reduction in surface expression of the α_M_β_2_-integrin required for stable/firm adhesion, explaining the reduced number of monocytes in the psoriatic joint, compared to RA (Neumüller et al., 2001). Akin to lymphocytes, pathological alterations in the expression of the β_2_-integrins by neutrophils and monocytes contribute to the synovial leukocyte composition in these inflammatory arthritides.

Synovial blood vascular endothelial cells (sEC) act as the gatekeepers to the joint. Crucially, Abbot et al. (1999) demonstrated that sEC from RA patients are imprinted with a disease-specific tissue memory and are in a pre-primed state that is maintained in culture. This pathogenic phenotype enables TNFα-stimulated rheumatoid sEC to support greater levels of neutrophil and T-cell capture from flow and induced stable adhesion (Abbot et al., 1999). Further research examining the phenotype of sEC through the evolution of inflammatory arthritides, across diseases and its impact on the inflammatory infiltrate is urgently required. Similarly, synovial fibroblasts from inflammatory arthritides are epigenetically imprinted with a pathogenic phenotype (Parsonage et al., 2005). For example, RA synovial fibroblasts support elevated levels of B-cell (Shimaoka et al., 1998; Burger et al., 2001) and T-cell (Bradfield et al., 2003) pseudoemperipolesis (sub-fibroblast migration) in a CXCL12 dependent manner compared to dermal fibroblasts. Further to this, peripheral blood CD4^+^ T-cells from RA patients exhibit higher expression of the α6 integrin subunit and were more efficiently captured from flow to the extracellular matrix components, laminin and fibronectin, than control cells (Haworth et al., 2008). These interactions are likely to be responsible for localizing CD4^+^ T-cells in the laminin bordered perivascular cuff in the rheumatoid joint (Haworth et al., 2008) – as observed by confocal microscopy on tissue sections. Whilst neutrophils from healthy controls and clinically active RA bind at similar levels to fibronectin *in vitro*, significantly fewer cells adhered when they were isolated from patients in clinical remission – these observations were linked to reductions in the expression of L-selectin and α_L_-integrin on neutrophils following anti-TNFα therapy (Dominical et al., 2011). These studies start to elucidate the molecular mechanisms responsible for leukocyte interactions in the subendothelial space, and how these are altered by drug-induced clinical remission.

The combination of altered adhesive and migratory properties of leukocytes and endothelial cells in patients with RA further amplifies the aberrant trafficking of inflammatory cells during disease – the critical question of which cell type becomes dysregulated first remains to be answered.

### 3-Dimensional Models

Over the last 20 years, considerable effort has been made to address the limitations of 2-D culture systems in an attempt to generate a more representative 3-D model of the tissue (McGettrick et al., 2012; Buckley and McGettrick, 2018), in which leukocyte trafficking under static and physiological flow conditions can be observed (Munir et al., 2015; Mcgettrick et al., 2017) (Figure 2F). As a result, it is clear that tissue-resident stromal cells communicate with the neighboring endothelial cells to generate “stromal address codes” for tissue-specific and disease-specific regulation of leukocyte trafficking (Parsonage et al., 2005). Crucially the absence of these signals results in lymphocyte migration being frustrated, such that they shuttle back and forth across the inflamed endothelial barrier, with few cells penetrating a 400 μm deep collagen gel (McGettrick et al., 2008; Jeffery et al., 2013). A series of studies using phase-contrast microscopy has revealed that primary synovial fibroblasts from patients with acutely resolving synovitis or primary dermal fibroblasts from patients with RA undergoing joint replacement surgery limit the ability of endothelial cells to recruit lymphocytes from flow - in effect acting in an anti-inflammatory manner (McGettrick et al., 2009; Filer et al., 2017). By contrast, synovial fibroblasts from patients with RA undergoing joint replacement surgery were able to stimulate the endothelium, such that they were able to recruit neutrophils and lymphocytes in the absence of any other exogenous cytokines - thus exerting a pro-inflammatory action (Rainger et al., 2001; Lally et al., 2005; Smith et al., 2008; McGettrick et al., 2009; Filer et al., 2017). In particular, this response was due to the trafficking and presentation of fibroblast-derived chemokines (CXCL5 and CXCL12) on the endothelial surface, and enhanced leukocyte capture mediated through P-selectin (neutrophils) and α_4_β_1_-integrin-VCAM-1 interactions (lymphocytes) (Lally et al., 2005; McGettrick et al., 2009). Interestingly the synovial fibroblasts from patients at the earliest phase of RA communicate with the endothelium in a manner that is distinct from fibroblasts isolated from resolving synovitis (i.e., they do not suppress cytokine-induced lymphocyte recruitment), and also from fibroblasts from patients with RA undergoing joint replacement surgery [i.e., they are unable to activate the endothelium to recruit leukocytes in the absence of cytokines (Filer et al., 2017)] – this crucially reveals that the cross-talk, and therefore the patterns of leukocyte trafficking in RA, evolve as the disease progresses and suggest that therapies targeting these processes should also change as the disease evolves. Furthermore, blocking either hydrocortisone or IL-6 was able to reverse the pro-inflammatory effects of fibroblasts from patients with RA who had to undergo joint replacement surgery (Lally et al., 2005; Smith et al., 2008; McGettrick et al., 2009), adding further evidence to the mode of action of glucocorticoid or tocilizumab therapy in these patients. Of note, all these studies focused on the interaction of fibroblasts from patients with inflammatory arthritis with endothelial cells isolated from healthy donors. Additional studies are now required to expand this further by incorporating patient-derived sEC’s that exhibit disease-specific phenotypes (as highlighted above) and to elucidate the impact this has on the ability of fibroblasts to regulate leukocyte trafficking. Furthermore, there is also a need to broaden the inflammatory arthritides from which cells are isolated to reveal those features of arthritis that are shared across a range of arthritides and those that are unique.

## Imaging Leukocyte Trafficking *in vivo* – Observational Studies

The regulation of leukocyte trafficking is multifactorial – involving blood flow; endothelial and stromal responses. Similarly, inflammatory arthritides can be systemic diseases, affecting multiple joints and other organ systems. The only way to truly understand how arthritic pathology influences the kinetics, spatial location and molecular mechanisms of leukocyte migration into, through and away from the joint is through *in vivo* analysis – either preclinical murine models of disease or imaging of patients joints. However, these approaches are significantly more complicated than those we have discussed above, due to the intricate nature of joints (Gompels et al., 2010). As a result, most studies to date have focused on mapping cellular movement, with few conducting mechanistic investigations.

Initial efforts to observe infiltration of human leukocytes movement in real-time began by metal-tagging purified leukocytes subsets and re-injecting them back into the subject, allowing the small gamma particles released by the tagged cells to be detected and tracked (Uno et al., 1986; Dudhia et al., 2015). Tagging human leukocytes with indium-111 (^111^In) revealed the accumulation of tagged cells in the joints of human patients with swelling and active RA, which were not observed in patients where swelling and pain were not present (Uno et al., 1986) or those with inactive disease (Uno et al., 1992). Moreover, anti-TNFα therapy reduced ^111^In-labeled granulocyte trafficking into patient joints over 22 h (Taylor et al., 2000). Techniques subsequently progressed to enable tagging of specific cell types, such as anti-T-cell (CD3) technetium-99m (^99*m*^Tc) tagged antibodies, which were observed accumulating in the moderately or severely painful joints in RA and PsA patients, but not in those joints where pain levels were low or absent (Marcus et al., 1994). Indeed, clinically active and inactive joints have also been identified using radiolabeled E-selectin in patients with RA (Chapman et al., 1994; Chapman et al., 1996; Jamar et al., 2002). Once again, anti-TNF-α therapy (adalimumab) decreased ^99*m*^Tc-labeled leukocyte accumulation into the joints of RA patients 2 weeks after the treatment onset, which subsequent studies suggested that this was due to increased ^99*m*^Tc monocyte/macrophage egress from the joint as numbers of monocytes entering remained unaffected by adalimumab treatment (Thurlings et al., 2009; Herenius et al., 2011). Similarly, metal tagged antibodies against E-selectin have also revealed its up-regulation in porcine arthritic joints but not control joints (Chapman et al., 1994). Thus linking the active movement of T-cells into the joint and changes in capture receptor expression with swelling, pain and disease activity experienced by the patients. Similarly, blood neutrophils tagged with technetium-99m hexametazime were observed ingressing into the rheumatoid joint over 22 h by gamma camera imaging, a response that was blocked by glucocorticoid therapy (methylprednisolone) (Youssef et al., 1996). By contrast, when synovial fluid neutrophils tagged with ^111^In were injected intra-articularly their egress from the synovial space into the periphery was unaffected by methylprednisolone treatment (Youssef et al., 1996). Further supporting the involvement of migrating neutrophils in the RA pathology, and the beneficial action of glucocorticoid therapy on limiting their movement/effector functions.

Positron emission tomography (PET) tracers allow a non-invasive means to measure cellular accumulation (White et al., 2017; Momcilovic et al., 2018) (Figure 2H). For instance, higher levels of the PET tracer [18F]F-AraG were detected in the arthritic paws of AIA mice, whilst no tracer was observed in control paws (Franc et al., 2017). Similarly, in RA and PsA patients, the [18F]-FDG PET tracer was shown to accumulate at sites of synovitis, but not in unaffected joints (Chaudhari et al., 2016). Thus, indicating an accumulation of activated immune cells in the arthritic joint, which could result from either increased leukocyte migration or enhanced proliferation of the cells within the tissue or a combination of both. Protein specific PET tracers, such as ^68^Ga-Aquibeprin or ^68^Ga-Avebetrin that bind specifically to α_5_β_1_-integrin or α_V_β_3_-integrin, respectively, can also be used to track the expression pattern of molecules involved in leukocyte trafficking (Notni et al., 2019). Such tracers showed increased expression of α_5_β_1_- and α_V_β_3_-integrins in arthritic joints compared to non-arthritic joints in mice with CIA (Notni et al., 2019), suggesting arthritis increases the expression of these integrins to facilitate leukocyte accumulation. Tracer studies are currently limited to detecting global cellular activity (accumulation/proliferation) but are unable to identify individual cells or subsets or their movement.

The greatest advancement in imaging the dynamics of leukocyte trafficking *in vivo* has come from the development of two-photon microscopy, which allows tissues, including the joints and blood vessels, of living organisms to be imaged in real-time enabling the observer to visualize specific parts of the adhesion cascade including velocities (Chen et al., 2017; Cai et al., 2019) (Figure 2I). For example, leukocyte rolling and adhesion to arthritic joints (Veihelmann et al., 1999), or neighboring tendons [achilles and patella (Gál et al., 2005)], increased in a time-dependent manner in the initial phases of the disease, after which the number (Veihelmann et al., 1999) and velocity (Gál et al., 2005) of rolling cells steadily declined, whilst the absolute numbers of adherent cells remained elevated in both AIA and proteoglycan induced arthritis (PGIA) models relative to the control animals. Moreover, rolling velocities increased at early time points (4 h) and absolute numbers of adherent leukocytes were reduced over the first 24 h in L-selectin knockout mice with AIA - observed using intravital microscopy of the synovial post-capillary venules (Szántó et al., 2004). Similarly, more LysM-eGFP granulocytic myeloid cells were observed moving at a much slower mean migration velocity through metatarsal tissue of AIA mice using intravital microscopy compared to controls (Byrne et al., 2012). Moreover, adoptively transferred fluorescently labeled antigen-presenting cells (APC) isolated from the spleen and draining lymph nodes of PGIA mice were detected inside the ankles of severe combined immunodeficient (SCID) mice by multiphoton imaging (Angyal et al., 2010). However, when splenic T-cells were transferred they were only detected in the lymph nodes, revealing low levels of T-cell migration directly into the joint following induction of PGIA (Angyal et al., 2010). Near-infrared whole animal imaging tracked the increased infiltration of F4/80^+^ monocyte/macrophages into arthritic joints over the first 6 h of AIA (Hansch et al., 2004). Taking this further, biofluorescence whole animal imaging studies revealed the more effective localization of anti-TNFα drugs (certolizumab pegol, adalimumab, and infliximab) to arthritic joints than non-arthritic joints in mice with CIA (Palframan et al., 2009).

Alternative strategies employing the use of bioluminescence, commonly involving luciferase, avoid the need for excitation of the label of interest (Figure 2J). For instance, Nakajima et al. (2001) tracked the localization of type-II collagen specific GFP-luciferase CD4 T-cell hybridomas following their injection into mice with CIA using whole animal real-time live bioluminescence imaging with the *in vivo* imaging system (IVIS) over a 7 days period (Nakajima et al., 2001). Initially GFP-luciferase CD4 T-cells were observed accumulating in the lungs after 24 h, but subsequently moved to the arthritic joints where the intensity of GFP-luciferase signal, and therefore the accumulation of cells, increased between 3 and 5 days and was still detectable at the same level 7 days post-injection (Nakajima et al., 2001). Using this technology, the locations of specific cell types can be observed throughout an experiment, however, it does not provide detailed data outlining specific cell numbers.

Advances in photoconvertible reporter mice (e.g., Kaede) are now allowing us to gain some insights into the migratory journey of leukocytes to and from tissues. For example, in Kaede mice, cells are converted from green to red fluorescence with a UV light, allowing researchers to track the movement of cells from the tissue of photoconversion into distance sites (Porter et al., 2018; Steele et al., 2019) (Figure 2K). On a technical note, it is crucial that tissues neighboring the photoconversion site are appropriately shielded from the light source to avoid converting these cells also. Whilst there are currently no publications reporting photoconversion of an inflamed joint or the neighboring draining lymph nodes we are aware of several groups working in this area and await the outcome of these studies. That said, groups have photoconverted areas of the small intestine and colon in arthritic mice (CIA and KRN, respectively) and detected converted cells, mainly CD4^+^ T-cells, in the joint and the draining lymph node between 1 (Morton et al., 2014) and 4 days (Tajik et al., 2020) later by flow cytometry. Combining these data with the earlier IHC studies from the late 1990s further confirms the importance of the ability of gut-derived leukocytes to migrate into arthritic joints in the pathogenesis of the disease.

Whilst advancements in imaging technologies allow the dynamics of leukocyte trafficking patterns or the expression profile of adhesion molecules and cytokines to be observed *in vivo*, we are not yet in the position to fully track these processes in patients over their disease and treatment journey. Improving imaging of molecular mechanistic changes, such as alterations in receptors and ligands, that occur as leukocytes enter and exit the joint is essential to fully understanding these processes and to identify targets to limit trafficking of pathogenic leukocytes whilst promoting the movement of regulatory leukocytes necessary to resolve the inflammatory response and repair the damaged joint.

## Summarizing Our Current Understanding of the Mechanisms Driving Inflammatory Arthritis

**FIGURE 3:**
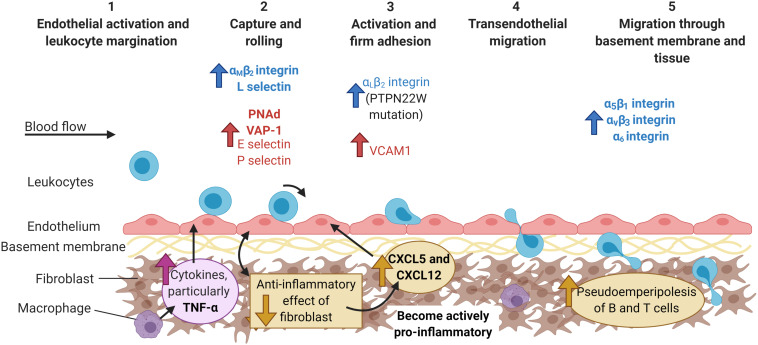
The multi-step adhesion cascade in chronically inflamed joints. The etiology of most inflammatory arthritides remains unknown. At some point during the initial phases of disease, the endothelial cells up-regulate the expression of the typical adhesion molecules (E and P selectin, VCAM), as well as other molecules usually found in HEV venules (PNAd) or the gut (VAP-1). Furthermore, fibroblasts switch from homeostatically anti-inflammatory, to actively pro-inflammatory with the production of chemokines (CXCL5 and CXCL12) that are shuttled to the endothelium. Leukocytes from arthritic patients exhibit higher expression of integrins, such as α_L_β_2_-, α_M_β_2_-, and α_6_-, and are recruited to the endothelium via P-selectin (neutrophils) and α_4_β_1_-integrin-VCAM-1 interactions (lymphocytes). Increased expression of α5β1, αvβ3, and α6 integrins on leukocytes aids their migration through the basement membrane and tissue, whilst fibroblast interactions with T and B cells increases pseudoemperipolesis. Molecules are colored according to cell type: leukocytes = blue and endothelial cells = red, and highlighted bold to denote major contributors to infiltration during inflammatory arthritis, but not in response to infection or damage. PNAd, peripheral node addressin; VAP-1, vascular adhesion protein 1; VCAM-1, vascular adhesion protein 1. Created with BioRender.com.

It is clear that patients with inflammatory arthritides, such as RA or PsA, have defects in one or more of the security checkpoints that normally regulate leukocyte trafficking (Buckley and McGettrick, 2018) (Figure 3). Firstly synovial endothelial cells (Abbot et al., 1999) and fibroblasts (Parsonage et al., 2005) are imprinted with a pathogenic pro-inflammatory phenotype that is maintained in culture, and which causes these cells to express elevated levels of proinflammatory and pro-recruitment mediators, such as adhesion molecules, chemokines, lipids and cytokines (Parsonage et al., 2005). Moreover, synovial endothelial cells acquire tissue-specific traits associated with mucosal endothelium (VAP-1) (Salmi et al., 1997) and high endothelial venules (PNAd) (Salmi et al., 1997; Cañete et al., 2007) allowing the aberrant trafficking of gut-homing and lymph node homing leukocytes to the joints, and in some cases the formation of tertiary lymphoid structures (Morton et al., 2014; Tajik et al., 2020). Synovial fibroblasts also display positional memory for their location within a joint (Croft et al., 2019; Wei et al., 2020), and differ across joints (Frank-Bertoncelj et al., 2017) – this adds further complexity to the regulation of leukocyte migration within the synovium itself, and for which newer imaging modalities are just starting to reveal insights. Finally, the bi-directional cross-talk between endothelial cells and fibroblasts evolves as the disease progresses (Filer et al., 2017), altering the composition of leukocytes in the joint over the disease history. Thus pathogenic changes in the joint tissue microenvironment actively support the aberrant leukocyte trafficking and accumulation, and so one strategy would be to reset the tissue microenvironment to reverse pathology. Yet, leukocytes from these patients can also display pro-adhesive, pro-migratory phenotypes, linked to genetics [e.g., PTPN22W (Bayley et al., 2015; Burn et al., 2016)], altered metabolism (Pucino et al., 2019, 2020) or protein expression [adiponectin receptors (Chimen et al., 2015); TSK5 (Shen et al., 2017)], which tend to lead to enhanced or prolonged α_L_β_2_-integrin expression (Yokota et al., 1995; Burn et al., 2016) facilitating their entry and retention within the inflamed joint. However, this raises the “chicken and egg” question of whether changes in the tissue or changes in leukocytes are the first to happen. Of note, we know much more about the molecular mechanism governing these processes in the context of RA, than other inflammatory arthritides.

Many imaging studies using a wide range of technologies have demonstrated the significant role TNFα plays in the initiation and propagation of inflammation in the joints of humans with RA (Barrera et al., 2001) and mice (Williams et al., 1992; Sudoł-Szopińska et al., 2013). Of relevance here is the reduction in leukocyte accumulation in arthritic joints, as well as changes in the expression of adhesion molecules [e.g., E-selectin and VCAM-1 (Tak et al., 1996)] and chemokines (e.g., IL-8) (Butler et al., 1995) in response to TNFα inhibitors. Despite this, not all RA patients respond to the various on the market, which might be explained, at least in part, by the distinct synovium leukocyte pathotypes observed in tissue sections from patients (Nerviani et al., 2020). Few, if any studies have examined leukocyte numbers or expression of key molecules involved in the recruitment cascade for other chemical or biological disease-modifying anti-rheumatic drugs (DMARDs). Understanding how DMARDs impact the key components and cells of the cascade will allow the mechanism of initiation, development and resolution of inflammatory arthritides to be identified, possibly leading to personalized medicine depending on disease stage.

## The Future of the *In Vitro* Joint

Technologies to make more *in vivo-like* constructs are continuing to vastly improve: ranging from 3-D self-organizing tissue culture organoids; through to microfluidic channels and building up to 3-D cell cultures incorporating microfluidic technology to create organs-on-chips, and finally the fabrication of tissue-like structures. All of these techniques have improved tremendously over the past 10 years, but their use in studies of leukocyte trafficking in the context of inflammatory arthritides remains limited (reviewed by Damerau and Gaber, 2020).

Given inflammatory arthritides are driven by multiple cell–cell interactions, 3-D organoids offer an excellent way of interrogating these communication pathways in more detail. Unfortunately, the 3-D nature of these constructs currently restricts the possibility of real-time live imaging, so these models are commonly analyzed upon sectioning as seen for whole tissue. For example, TNFα induced the self-organization and proliferation of a 3-D micromass of fibroblast-like synoviocytes from RA patients into the two distinct layers found in the joint (a lining and sublining layer), implying fibroblasts maintain positional memory in culture enabling them to organize themselves as seen in the joint (Calvo et al., 2017). Building upon this, organoids consisting of fibroblasts and endothelial cells suspended in a matrigel droplet revealed that up-regulation of NOTCH3 ligands on the vasculature help to drive the spatial organization of fibroblasts into lining and sublining layers, where deletion of NOTCH3 reduced the clinical score and inflammatory infiltrated observed by H&E in mice with STIA (Wei et al., 2020). Crucially a subpopulation of synovial fibroblast (fibroblast activation protein α+; FAPα+) is responsible for driving leukocyte infiltration in STIA mice, where deleting FAPα+ fibroblasts reduced leukocyte numbers in the arthritic joint (Croft et al., 2019). Collectively these studies highlight the importance of fibroblast subtypes in regulating leukocyte trafficking and the ability of endothelial cells to influence the phenotype of the neighboring stroma, but crucially they demonstrate the possibility of modeling the human diseased joint using fibroblast-endothelial cell 3-D organoids *in vitro*. Of note, tumor and mural organoid models incorporating mesodermal progenitor cells were able to form a hierarchical structure of blood vessels (Wörsdörfer et al., 2019), suggesting the same could be done and used to model the vasculature in inflammatory arthritides.

Advances in 3-D printing are driving forward the field of microfluidic modeling of leukocyte recruitment in a disease context (Sackmann et al., 2012; Venugopal Menon et al., 2018), where incorporating precious, but limited, patient material into high-throughput mechanistic screening studies is becoming a reality. Such systems have effectively mimicked the disturbed flow patterns seen in vessel bifurcations, highlighting these as areas where endothelial cells can support the recruitment of the monocyte cell line, THP-1 – as seen in atherosclerosis (Khan and Sefton, 2011). Hence microfluidic channels may help to elucidate the disparities seen in the vascular patterns in patients with PsA and RA (Kennedy et al., 2010). Addition of collagen scaffolds; tuneable chemical gradients (Wu et al., 2017) and stromal cells, to the microfluidic channels are paving the way for the development of personalized “organs-on-a-chip” which present the possibility of precision medicine (Van Den Berg et al., 2019). In the context of inflammatory arthritis, microfluidic systems have been used to track the migration of the cadherin-11 expressing synovial cell line (SW982) toward an activated osteoclast cell line (RAW264.7), where the co-culture construct enhanced SW982 migration and osteoclast activity compared to the monocultures (Ma et al., 2018). Moreover, 3-D “synovium-on-a-chip” with an integrated time-resolved light scatter biosensor has been generated that allows the visualization of TNFα induced fibroblast organization into lining and sublining layers over 2 days (Rothbauer et al., 2020) (e.g., Figure 2G). Incorporating endothelial cells and leukocytes into such models would enable *in vivo* like analysis of recruitment and it is most likely only a matter of time before these are generated. Furthermore, other *in vivo* like constructs are being formed, such as the living vascular tissue fabricated by direct culture of collagen, smooth muscle cells and endothelial cells, presenting another way to move away from *in vivo* models, whilst accessing leukocyte infiltration (Meghezi et al., 2015). These studies highlight how rapidly microfluidic and *in vivo* models are improving, providing the opportunity to access multiple cell:cell interactions, whilst also mimicking physiological aspects such as blood flow. This is crucial in understanding inflammatory diseases such as RA, PsA and IA, which are known to be driven by uncontrolled leukocyte recruitment, but exactly how and why this occurs is yet to be elucidated.

The era of precision medicine for inflammatory arthritis is fast approaching (reviewed by Aletaha, 2020; Miyagawa and Tanaka, 2020) with “big data” providing insights into different patient populations and further subgrouping patients according to their underlying process driven pathology – yet imaging remains crucial for initial discoveries and confirming outputs of omics analysis (e.g., Lewis et al., 2019). Indeed, the use of synovial tissue signatures improves the prediction that a patient will require biological therapy 12 months after diagnosis (Lliso-Ribera et al., 2019). Furthermore, advances in spatial transcriptomics now enables mapping of gene expression profiles onto images of tissues, providing for the first-time spatial context to gene expression data (Burgess, 2019). Such technology has revealed that central memory T-cells were dominant in RA synovial tissue sections, whilst in SpA tissues effector memory T-cells were most prominent (Carlberg et al., 2019), and thus will enable more targeted therapies to be delivered to these patients in the future. Advances in imaging technologies, in particular the “synovium-on-a-chip” described above, incorporating patient synovial material provides the opportunity to pre-screen possible treatment options based on identified “patient specific signatures,” ultimately offering the realistic hope of achieving precision medicine for all patients based on their cellular and molecular processes driving their disease pathology.

## Conclusion

Significant advances in imaging modalities over the last 60 years have vastly improved our understanding of leukocyte infiltrates in the inflamed joints in numerous inflammatory arthritides in patients as well as *in vitro/in vivo* models. It is evidently clear that the local microenvironment of an inflamed joint dictates the spatiotemporal dynamics of leukocyte entry and egress, and it is highly likely this differs in different regions of a given joint, between patients, as the disease evolves and in response to treatments. Despite our advances, there is still much we do not fully understand particularly in the context of human disease, and for that further advances are needed to image these processes directly in patients but more realistically to enable the imaging of complex *in vitro* constructs of a patient’s joint microenvironment. Only then will we be able to truly map the spatiotemporal dynamics and molecular mechanisms for a patient at a given time point in their disease history. Ultimately the aim would be to develop drug combinations that can limit the trafficking of pathogenic effector leukocytes whilst promoting the function and presence of regulatory leukocytes to switch off the inflammatory response and induce clinical remission/cure the disease.

## Author Contributions

All authors listed have made a substantial, direct and intellectual contribution to the work, and approved it for publication.

## Conflict of Interest

JM’s Ph.D. studentship was partially funded by Novartis. The remaining authors declare that the research was conducted in the absence of any commercial or financial relationships that could be construed as a potential conflict of interest.
